# *In vivo* evaluation of three-dimensional of volumetric changes using a CAD/CAM chair-side system: Technical procedure

**DOI:** 10.4317/jced.53531

**Published:** 2017-03-01

**Authors:** Rubén Agustín-Panadero, Alberto Ferreiroa, Agustín Pascual-Moscardó, Antonio Fons-Font, María-Fernanda Solá-Ruíz

**Affiliations:** 1 DMD, PhD, Associate Professor. Department of Dental Medicine, Faculty of Medicine and Dentistry, University of Valencia, Spain; 2 DMD, PhD, Associate Professor. Department of Buccofacial Prosthetics, Faculty of Dentistry, Complutense University of Madrid, Spain; 3 MD, DMD, PhD, Professor. Department of Dental Medicine, Faculty of Medicine and Dentistry, University of Valencia, Spain; 4 MD, DMD, PhD, Adjunct Professor. Department of Dental Medicine, Faculty of Medicine and Dentistry, University of Valencia, Spain

## Abstract

An intraoral digital scanner in combination with specialized three-dimensional surface analysis software monitors volumetric changes to soft tissues or dental restorations. This technology can evaluate the success of a specific technique or medium- or long-term clinical outcomes in both clinical and research situations. This article describes how this technology was used to provide immediate chair-side data analysis without the help of specialized laboratory support.

** Key words:**Intraoral scanner, CAD-CAM, best fit-method, surface tessellation language.

## Introduction

The outcomes of clinical procedures in dentistry are usually analyzed by making baseline evaluations and comparing them with evaluations obtained at a series of follow-up appointments; this determines the changes produced over time and the results of each specific treatment (1,2).

To determine these changes quantitatively, digital technologies represent an important advance for both visual analysis and metric evaluation of changes to soft tissues, teeth, or restorations. Specialized software is used to generate surface tessellation language (STL) files corresponding to the preparation, restoration, soft or hard tissues, which can be superimposed over one another, computing all possible orientations, and selecting the one with the best object-to-object penetration. This type of procedure or function is known as the best-fit-method (3-5).

To generate STL files, the first step is to take a conventional impression and cast it, and the second step is to scan the master cast with an extraoral scanner (6). But STL files can also be obtained directly from the patient’s mouth taking a digital impression with an intraoral digital scanner (7-9). Several intraoral digital scanners are available to clinicians; they are based on different technologies for information capture including wavefront sampling technology, confocal microscopy technology, or active triangulation technology with blue light (10,11). These devices not only capture data, but can also perform comparative analysis of different STL files by means of the best-fit-method, using software installed in the scanning unit itself. This allows the clinician to carry out the procedure immediately without sending data away to a specialized laboratory for analysis (12).

When it comes to analyzing three-dimensional surface data, this type of software offers clinicians and researchers advantages of speed and precision, and reduced economic cost, which are particular helpful for *in vivo* research purposes.

The aim of this article was to describe the application of analysis software used in conjunction with an intraoral digital scanner as a clinical tool for *in vivo* quantitative analysis of volumetric changes in a specific case.

## Case Report

**Figure 1:**
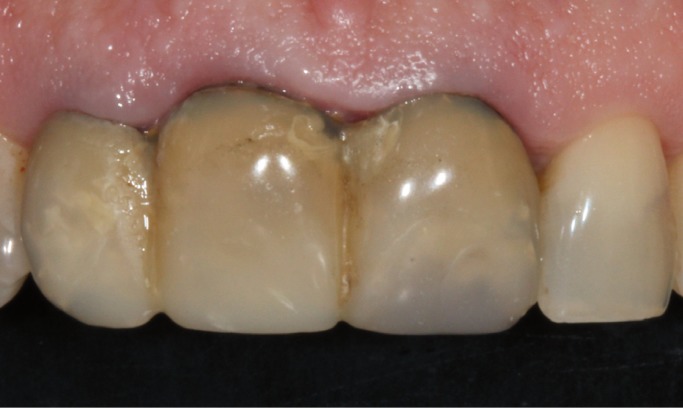
Initial situation of the patient with fixed dental prosthesis: inadequate aesthetics.

**Figure 2:**
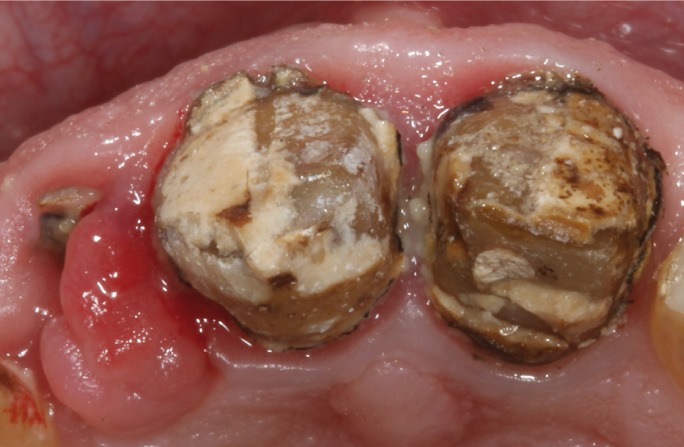
Occlusal view of the teeth after removal of the previous fixed dental prosthesis.

**Figure 3:**
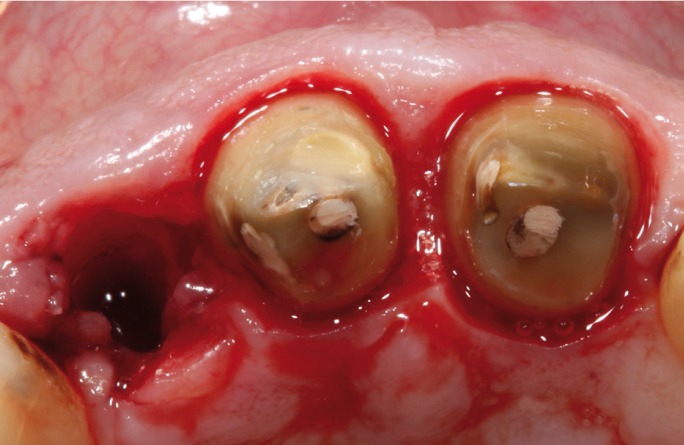
Occlusal view after extraction of tooth 1.2 and dental preparation of teeth 1.1 and 2.1 following BOPT protocol.

**Figure 4:**
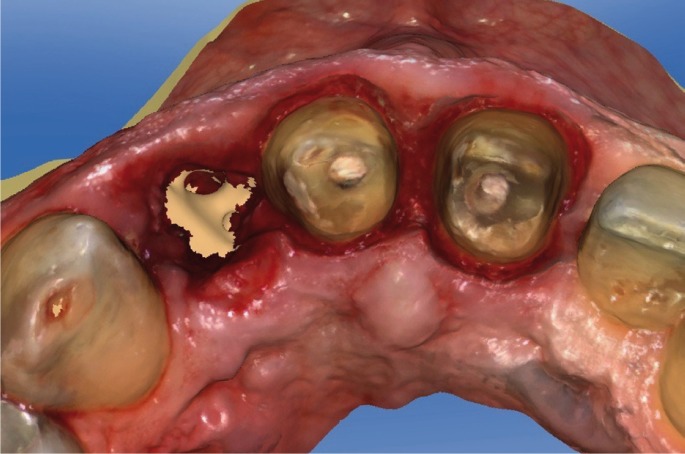
STL file occlusal view after extraction of tooth 1.2 and dental preparation of teeth 1.1 and 2.1 obtained from intraoral digital impression.

**Figure 5:**
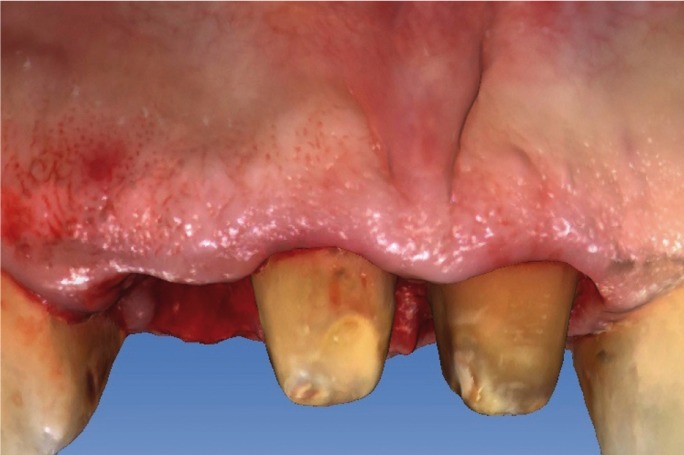
STL file buccal view after extraction of tooth 1.2 and dental preparation of teeth 1.1 and 2.1 obtained from intraoral digital impression.

**Figure 6:**
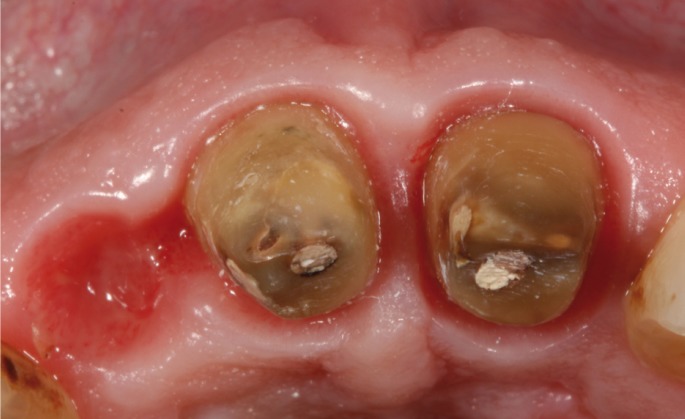
Occlusal View after 3 months.

**Figure 7:**
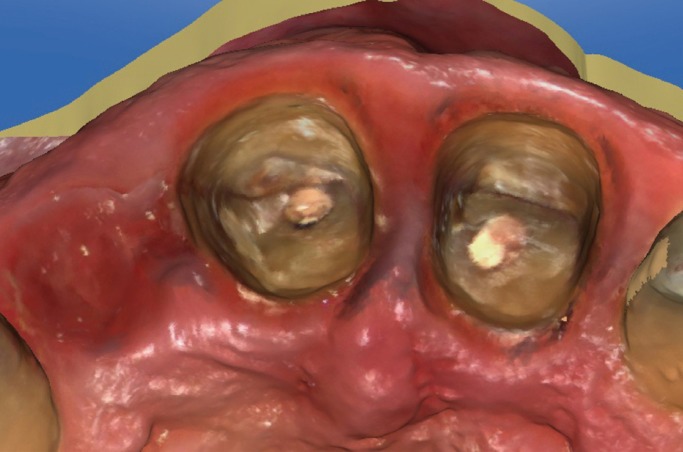
STL file occlusal view after three months.

**Figure 8:**
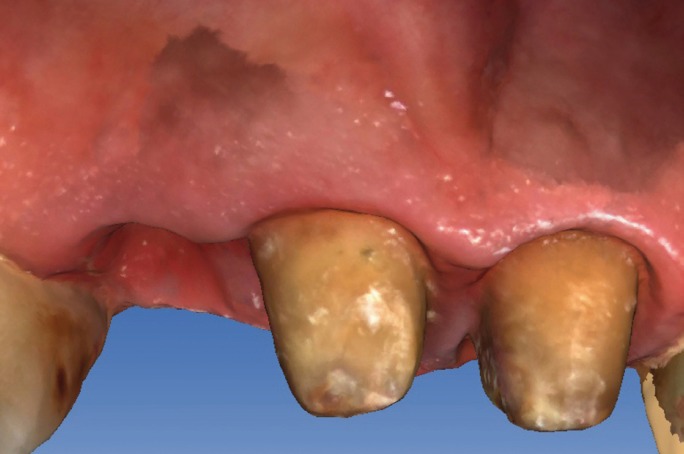
STL file buccal view after three months.

A 50-year-old male attended in prosthodontic teaching unit at Valencia University. Intraoral examination found that the patient had three metal-ceramic crowns at teeth 1.2, 1.1 and 2.1 with major misfit between the dental abutments and the crowns (Fig. 1). It was decided to remove the three crowns; an important caries lesion was observed on tooth 1.2 (Fig. 2). Finally, tooth 1.2 was extracted and tooth 1.1 and 2.1 were prepared following the biologically oriented preparation technique (BOPT)13-15 for preparing dental abutments to support a bridge from tooth 1.2 to 2.1 (Fig. 3). To determine volumetric changes, an intraoral digital scanner (Cerec Omnicam; Sirona) was used to generate STL files. The patient was scanned twice, first after dental preparation and extraction of tooth 1.2 (Figs. 4,5) and a second time 3 months later, before cementing the final restoration (Figs. 6-8). Both STL files were exported to analysis software (Oracheck; Cyfex) for clinical evaluation of volumetric changes to the soft tissues; this procedure could be carried out immediately during the same appointment.

In all cases, software analysis produces initial STL case files at baseline and then further STL files are generated at follow-up appointments. The different STL files can be combined and compared to evaluate volumetric changes to soft tissues. A selection of areas in the arrangement phase is rarely necessary because the software overlays automatically with precision. Nevertheless, it is possible to mark individual areas, which can be helpful in situations in which the two models to be overlaid exhibit major deviations. In the present case, differences between the 4-week and 3-month follow-ups did not present large deviations, and so it was possible for the software to superimpose data automatically.

**Figure 9:**
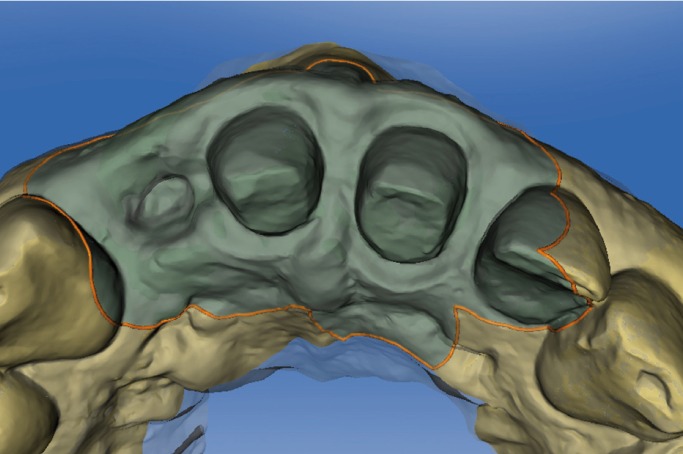
Defined study areas. The clinician has selected this area for comparison of STL files generated at different times.

**Figure 10:**
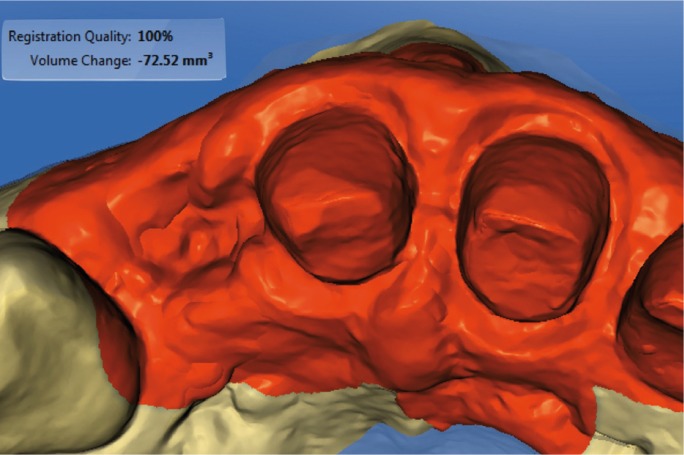
Volumetric differences in selected area.

**Figure 11:**
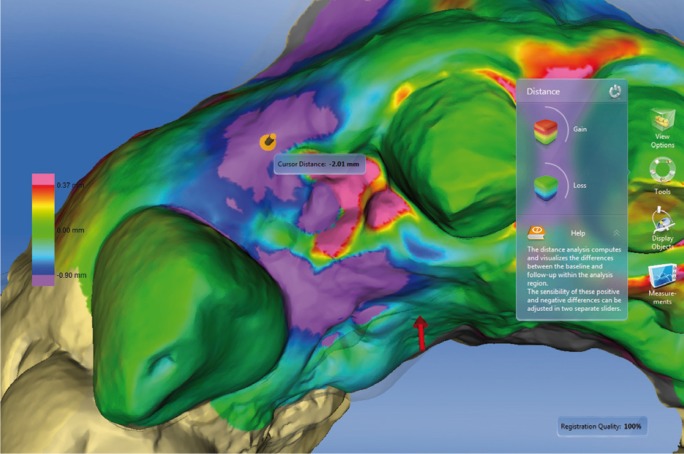
Distance between two STL files with color-coded scale. Violet color indicates a loss of volume. Here the loss of volume coincides with the extraction area. Red and pink indicates volume gain of the papilla between tooth 1.1 and 2.1.

A specific study region can be defined, defining an area of interest and then making comparisons STL files over time (Fig. 9). The software can make a volumetric analysis calculating the differences between STL files and differences between areas (Fig. 10). It is also possible to study differences in distances between STL files, which use a system of color-coding color to differentiate bet-ween positive or negative changes in distance (Fig. 11). This aims to analyze changes to distances between the grid points from one STL file to another.

**Figure 12:**
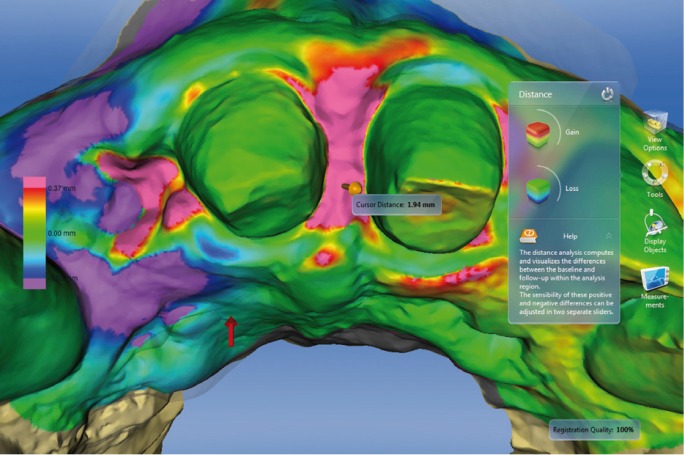
Distance between two STL files with color-coded scale. Red and pink indicates final volume increases in the papilla between the two upper central incisors.

**Figure 13:**
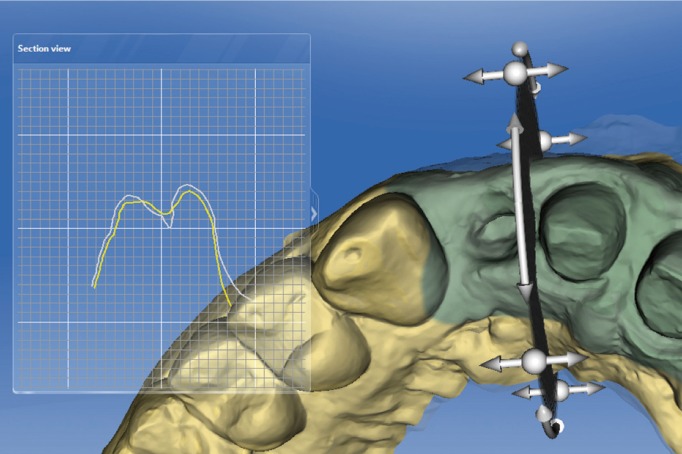
2-D cross-sections showing differences between two models.

As another analytic option, the software can be used to generate section views in two dimensions, showing differences between baseline and follow-ups. This enables metric measurements to be taken between the overlaid models (Figs. 12,13).

**Figure 14:**
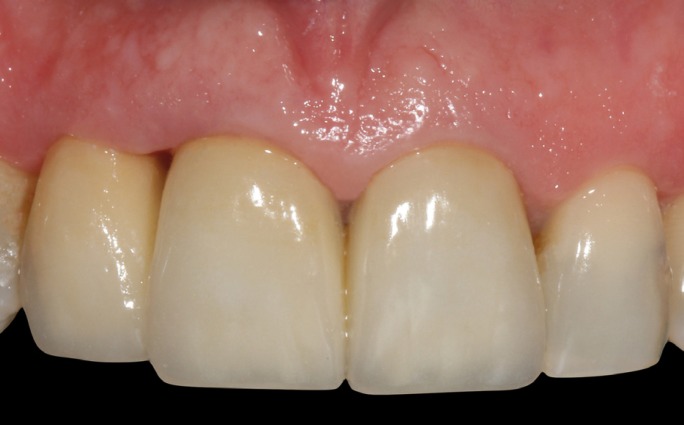
Final case outcome.

The final fixed dental prosthesis (FPD) was fabricated from zirconia ceramic. For cementation, the internal surface of the zirconia was prepared with tribochemical silica coating, first sandblasting the surface with 30-micron Al2O3 particles (Cojet Sand; 3M espe). The teeth were prepared with 35% phosphoric acid for 40 seconds and the FPD was cemented with dual-polymerized resin cement (Relyx Unicem 2 automix; 3M espe) (Fig. 14). Written informed consent was obtained from the patient.

## Discussion

This article describes the comparative analysis of initial and follow-up situations of a patient undergoing prosthodontic treatment, monitoring changes to soft tissue volumes. Comparative analysis was carried out using an intraoral digital scanner and specific software for applying a best-fit method to provide full data analysis immediately in a chair-side situation.

Using STL files, several research initiatives (1-5) have compared different situations over time; these include the positions of implants, comparing master casts obtained from intraoral digital impressions and conventional impressions with elastomers; changes to the volume of soft tissues after surgical procedures; or volumetric changes to dental restorations after different observation periods. STL files generated at different follow-up times are combined using specialized software such as Geomagic (Geomagic; 3Dsystems), normally used in industrial engineering (6). With this type of software, the STL files must be sent away to a center or laboratory for analysis carried out by an experienced operator (6). So whenever it is necessary to combine different files using a best fit-method, comparative research will require time and financial resources. But in the present case -thanks to Oracheck- the clinician could make an immediate comparative analysis of STL files, comparing the baseline model with follow-up STL files, without help from a specialized technician. This technology offers major advantages for conducting clinical studies in a range of fields/disciplines, as the clinician can make precise comparisons more quickly and efficiently (7-9).

In dental clinical situations, as described above, this type of software allows the clinician to make an objective assessment of changes to soft tissues, and enables him/her to forecast any need for changes to the interim restorations or for surgical treatments to improve the aesthetic integration of the gingiva with the final restoration.

## Conclusions

An intraoral digital scanner in combination with specialized three-dimensional surface analysis software will superimpose information registered at different points in time, which constitutes an ideal tool for the objective analysis of treatment outcomes related not only to soft tissue changes but also to changes to the dental restorations themselves. Such tools are useful in both clinical and research practice.
